# Subtrochanteric Fracture of the Femur Accompanying Pre-existing Ipsilateral Osteoarthritis of the Hip Successfully Treated with Intramedullary Nailing in the Lateral Decubitus Position: A Case Report

**DOI:** 10.7759/cureus.3081

**Published:** 2018-07-31

**Authors:** Toru Iga, Ken Kato, Tatsuro Karita

**Affiliations:** 1 Orthopaedic Surgery, Tokyo Metropolitan Tama Medical Center, Tokyo, JPN; 2 Orthopedic Surgery, Yokohama Rosai Hospital, Yokohama, JPN; 3 Orthopaedics, Tokyo Metropolitan Tama Medical Center, Tokyo, JPN

**Keywords:** subtrochanteric fractures of the femur, intramedullary nailing, surgical positioning, osteoarthritis of the hip joint, operating tables

## Abstract

A subtrochanteric fracture of the femur accompanying pre-existing osteoarthritis of the ipsilateral hip is rare. A deformity of the hip joint complicates the insertion of the intramedullary nail and varus malreduction is anticipated when surgery is performed on a fracture table with a perineal post. We report a successful case of intramedullary fixation performed in the lateral decubitus position and discuss the importance of avoiding varus and the superiority of the lateral position in surgery.

## Introduction

A subtrochanteric fracture of the femur accompanying pre-existing osteoarthritis of the ipsilateral hip (OA/STF) is rare [1-2]. In general, intramedullary nailing is the preferred method of treatment of subtrochanteric femur fractures [3-4]. However, arthritic deformities, such as the medial migration of the head and the shortening of the neck, complicate the correct placement of the entry point and the insertion of the nail along the long femoral axis. The entry point is often placed too far laterally when a fracture table with a perineal post is used. As a result, the fracture is reduced in varus malalignment [5], which must be avoided to prevent nonunion [3,6]. Our aim was to report a case of OA/STF and demonstrate the superiority of the lateral decubitus position for avoiding varus malreduction in intramedullary nailing.

## Case presentation

**Figure 1:**
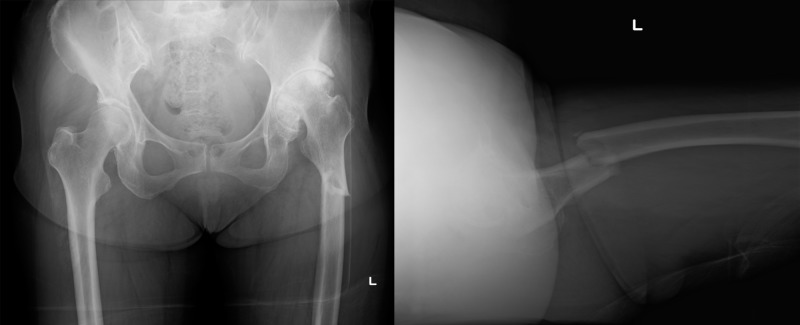
Preoperative radiography A subtrochanteric fracture of the left femur was seen. Cortical thickness, beaking of the lateral cortex, and a non-comminuted fracture met the criteria for an atypical fracture of the femur. The radiograph also showed osteoarthritis of the left hip with superomedial migration, external rotation of the femoral head, and shortening of the femoral neck, suggesting a difficulty in inserting a femoral nail.

A 72-year-old female presented to the emergency room with the complaint of left thigh pain and the inability to walk after tumbling. She had been suffering from osteoarthritis of the left hip but was able to walk with a cane and sit on a couch. She had also received the diagnosis of osteoporosis and had been taking alendronate 35 mg weekly for six years. The initial radiograph in the emergency room showed a non-comminuted subtrochanteric fracture of the left femur with cortical thickness and the beaking of the lateral cortex at the fracture site. The fracture line was transverse on the lateral side and oblique on the medial side. The radiographic findings and the weakness of the force that caused the fracture satisfied the criteria for an atypical fracture of the femur. The radiograph showed Kellgren-Lawrence grade 4 osteoarthritis of the left hip as well, with superomedial migration, the external rotation of the femoral head, and the shortening of the femoral neck (Figure 1).

Osteosynthesis with intramedullary nailing was planned. However, the deformity of the hip joint appeared to preclude correct nail insertion. Furthermore, whenever we used a fracture table in the past, we often observed that the perineal post of the fracture table impeded the adduction of the proximal fragment of the subtrochanteric fracture while the whole leg was being adducted, resulting in varus malalignment. On the other hand, we were aware of easy access to the entry point via the standard lateral decubitus position without using the perineal post. For these reasons, we decided to fix the fracture with a nail in the lateral decubitus position. Total hip arthroplasty with fracture fixation was not chosen because of its invasiveness, which was inappropriate for the rather mild, pre-injury symptoms due to osteoarthritis, and because of the patient’s wish to preserve the femoral head.

**Figure 2:**
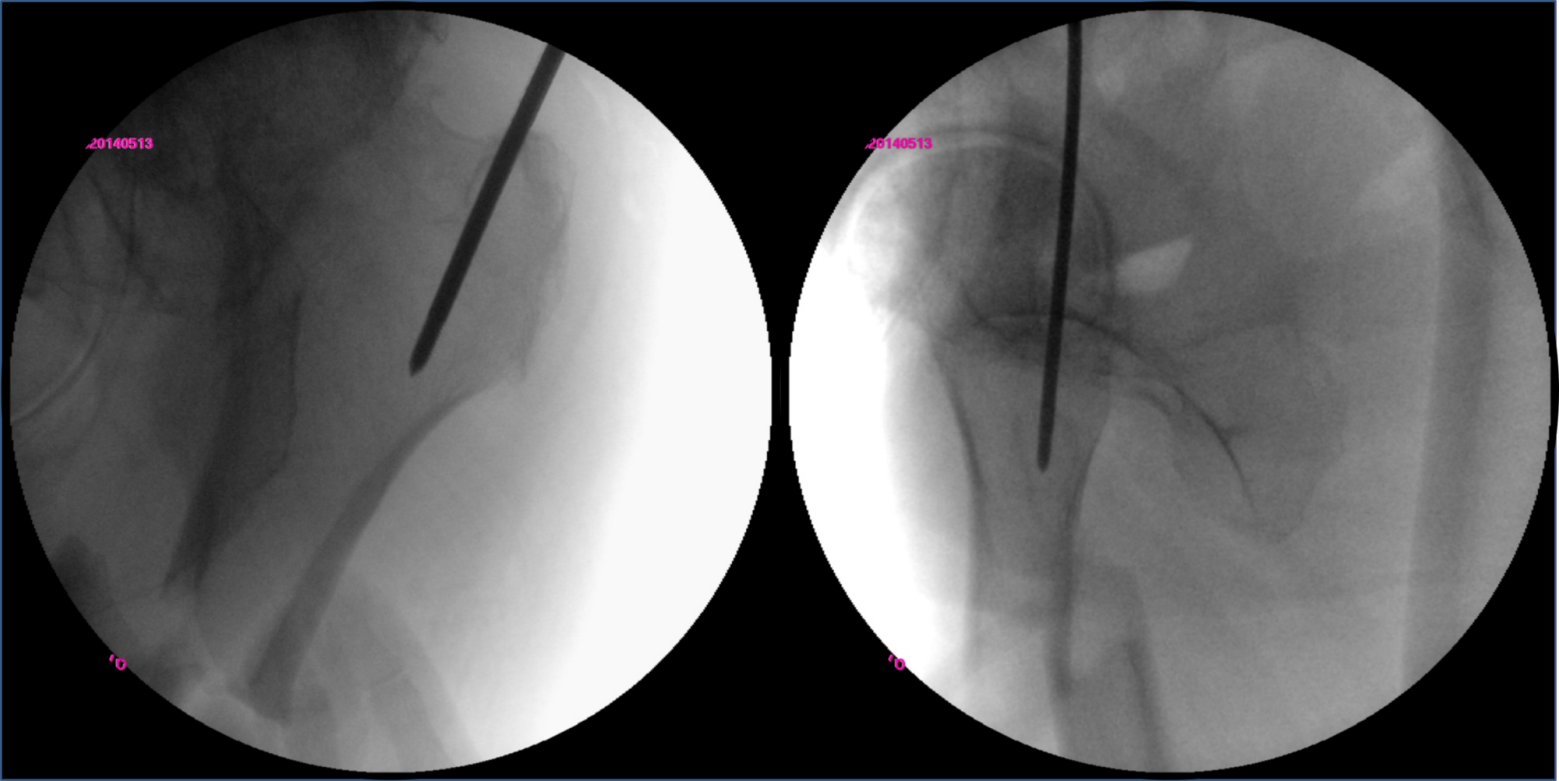
Fluoroscopic view of establishing the entry point An accurate entry point was easily accessed with the lateral decubitus position despite the deformity of the ipsilateral hip joint.

In surgery, a flat, radiolucent operating table was used. The whole injured leg was sterilized and draped, and the hip was slightly flexed. The C-arm was placed at the ventral side of the patient. Although the proximal fragment was externally rotated, the entry point was located easily by adjusting the C-arm to obtain a correct A-P view. A guide pin was inserted in line with the long axis of the proximal fragment to the level of the fracture without any interference by the torso (Figure 2).

**Figure 3:**
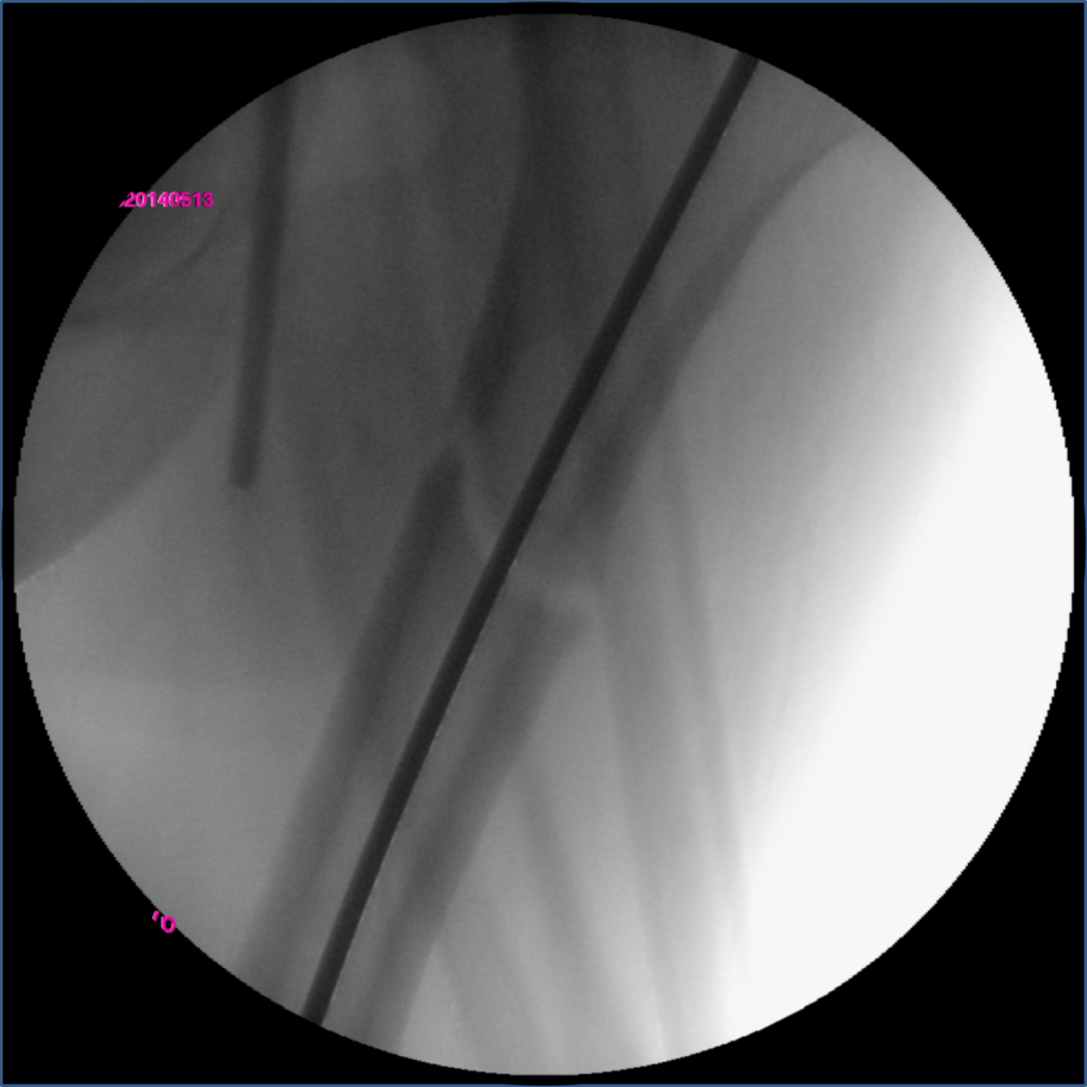
Fluoroscopic view of fracture reduction and guide pin insertion Coronal plane reduction was easy with the lateral decubitus position.

Then, the fracture was reduced with gentle manual traction of the thigh followed by the distal insertion of the guide pin. At this time, the fracture was perfectly aligned (Figure 3).

**Figure 4:**
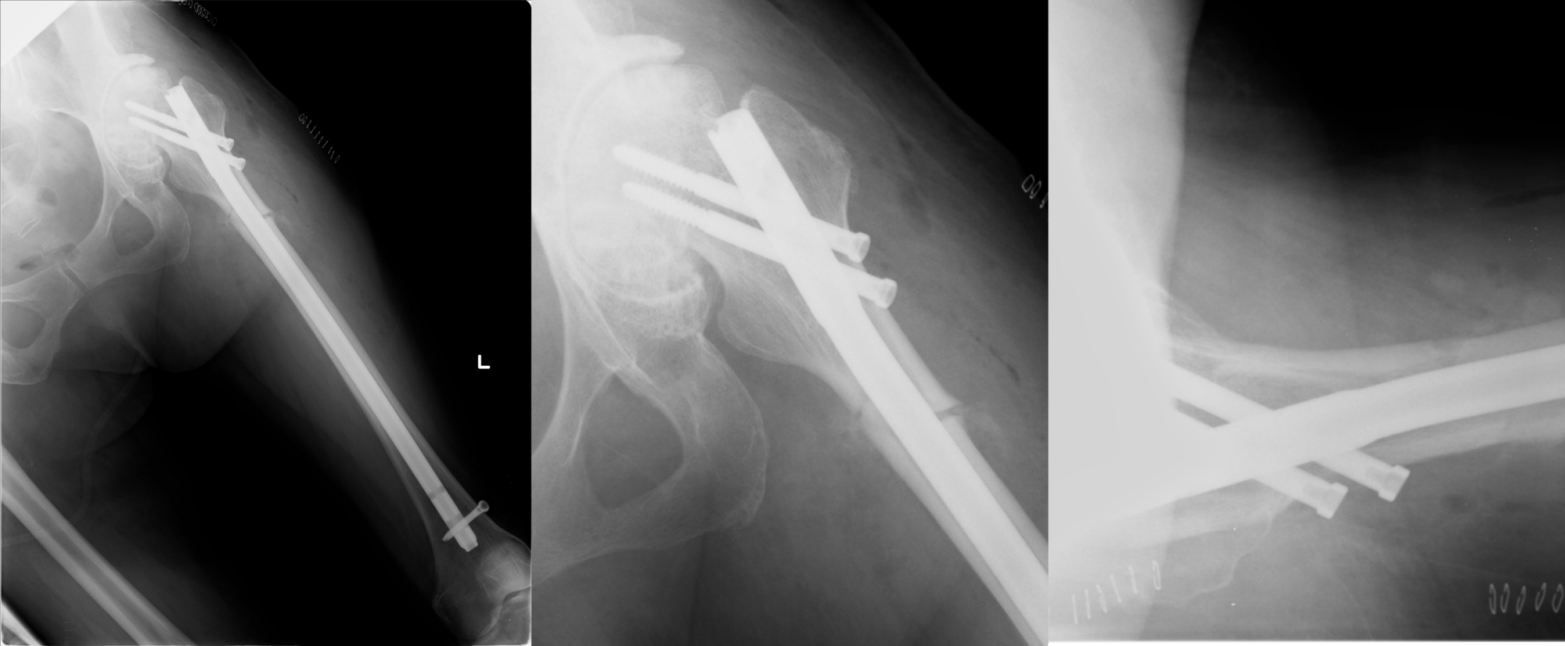
Postoperative radiography The fracture was anatomically reduced and fixed with a reconstruction nail.

 After reaming, a trochanteric-entry cephalomedullary nail (Trigen Trochanteric Antegrade Nail, Smith & Nephew, Massachusetts, US), 11.5 mm in diameter, was inserted, again, without any obstruction by the torso. After assuring rotational alignment, two cephalomedullary screws and a distal interlocking screw were inserted. Accurate, non-varus reduction was confirmed by a postoperative radiograph (Figure 4).

**Figure 5:**
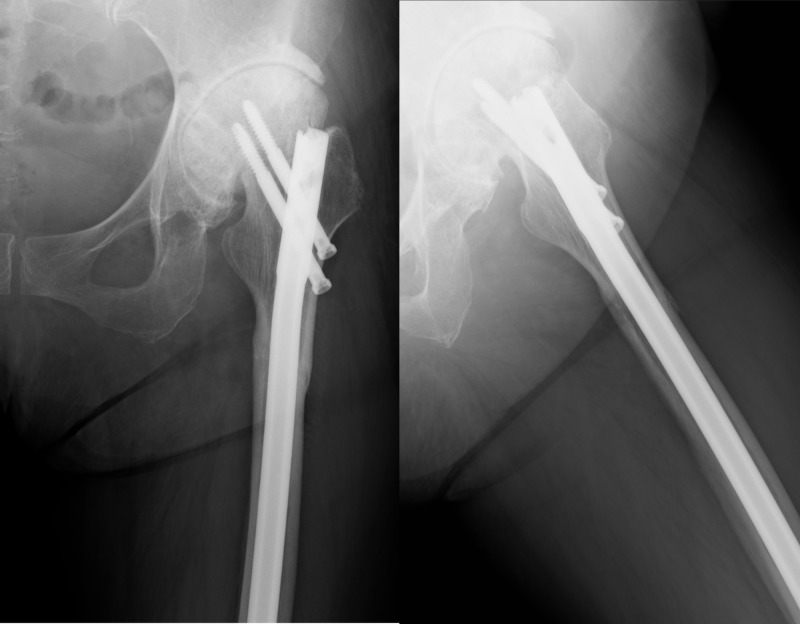
Solid union was confirmed 10 months postoperatively

The operating time was 122 minutes. The total blood loss was 86 ml. Toe-touch weight bearing was initiated soon and full weight bearing was allowed in five weeks. A solid union was confirmed by radiograph after 10 months (Figure 5). She was able to walk with a cane as she did before her injury without a worsening of the pain. Alendronate was discontinued after the injury and replaced with vitamin D supplements. No anabolic agent, such as teriparatide, was administered.

## Discussion

Subtrochanteric fractures of the femur complicated by osteoarthritis of the ipsilateral hip are uncommon and only found in a few reports [1-2]. However, they may become more common as the number of atypical subtrochanteric femoral fractures caused by the widespread use of bisphosphonates increases [7]. Understanding the proper surgical technique is necessary despite the rarity of this condition.

Surgical treatment for OA/STF is controversial. Osteosynthesis with an intramedullary nail is indicated for younger patients with mild pre-existing symptoms due to osteoarthritis. In such cases, arthroplasty is not preferred because of extensile exposure, increased morbidity, and an increased risk of revision surgery [2]. However, osteosynthesis without addressing the arthritic hip is questionable, especially for the elderly, since reconstruction using a cable-plate followed by arthroplasty with a long stem provides early functional recovery. Other reasons against using osteosynthesis with an intramedullary nail are the difficulty in gaining a correct entry point and the possibility of malunion, which may make future long-stemmed arthroplasty complicated [1]. In general, either osteosynthesis or arthroplasty can be selected, depending on the patient’s condition. We feel that osteosynthesis may be preferable for patients with mild pre-injury symptoms regardless of the radiographic severity of the osteoarthritis. Osteosynthesis with both intramedullary nailing and plating has satisfactory results. However, considering the frequent delayed union or nonunion observed in atypical femoral fractures, intramedullary nailing is preferable due to its mechanical superiority and endochondral repair. Plating for atypical femoral fractures is not recommended because of its unacceptably high failure rate [7].

When operating on a subtrochanteric fracture of the femur with intramedullary nailing, the entry point needs to be chosen correctly to avoid malreduction. If the entry point is misplaced only a few millimeters laterally, the fracture is angulated into varus, as the nail is inserted distally even if it was well-aligned before nail insertion [5]. Varus malalignment increases the natural tension-compression stresses on the subtrochanteric region and disturbs fracture union as well [3]. In fact, varus malalignment was observed in most cases of subtrochanteric nonunion with a breakage of the intramedullary nail [6]. Therefore, varus malreduction must be avoided. However, subtrochanteric fractures are often reduced in varus [4], especially when a patient lies supine on a fracture table where the injured leg has to be adducted in order to insert the nail [3,8]. However, the proximal fragment tends not to follow this movement because it is blocked by the perineal post. It should be noted that a perineal post is positioned adjacent to the fracture, not to the hip joint, and works as a pivot point for varus angulation [3]. Additionally, the working space is limited by the torso, especially in obese patients. Without enough space, a guide pin or a nail tends to be inserted in the distal-medial direction rather than along the long axis of the proximal fragment [8]. To make space, the torso is often moved to the contralateral side, but then the proximal fragment may be abducted by hip abductor muscles. Under these conditions, the entry point tends to be misplaced laterally. Thus, using a fracture table in intramedullary nailing for a subtrochanteric fracture poses the risk of varus malreduction. In addition, if there is any arthritic change in the hip joint such as central migration or collapse of the head or shortening of the neck, the entry point will be located more medially than in a normal hip joint, and access to the correct entry point will be limited [1-2]. Therefore, surgical positioning using some other means besides a fracture table with a perineal post is essential in intramedullary nailing for OA/STF.

**Figure 6:**
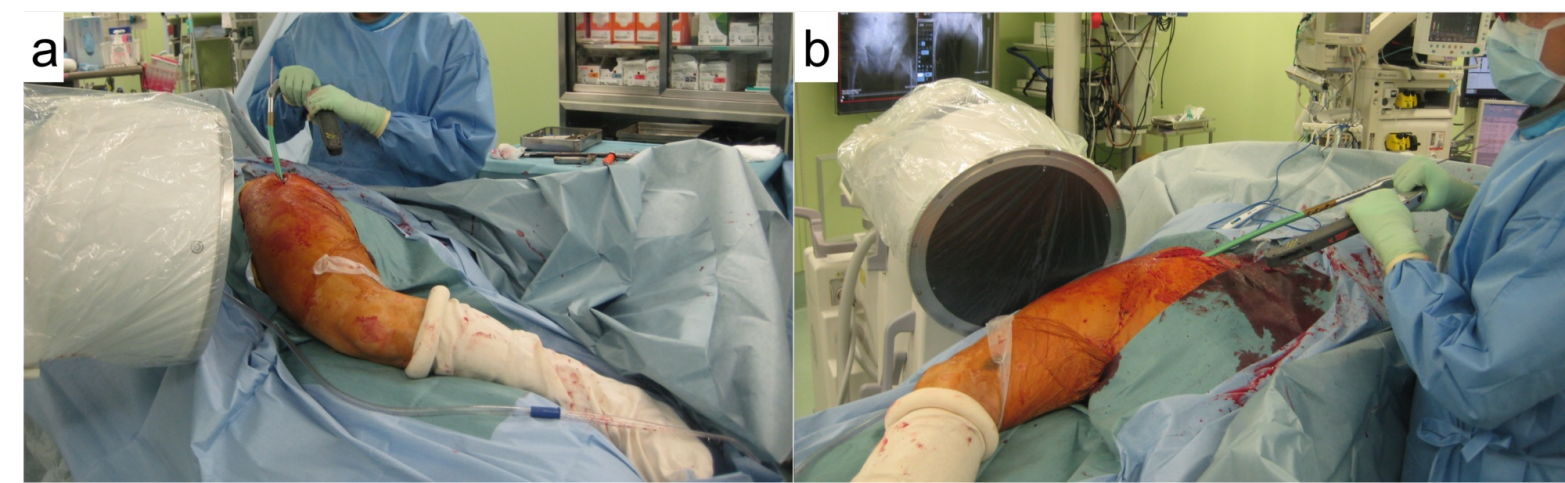
Inserting an intramedullary nail with the lateral decubitus position The injured hip is flexed in order to obtain sufficient working space for inserting the nail. A guide pin or a nail can be inserted without interference by the torso. This position also helps to reduce sagittal deformity. Note the setting of the fluoroscopy.

Intramedullary nailing in the lateral decubitus position on a standard radiolucent table for subtrochanteric fractures of the femur reportedly has satisfactory results [3,9-10]. In terms of coronal plane reduction, subtrochanteric fractures may sometimes drift into the valgus before reduction due to gravity [9], indicating that the adduction of the proximal fragment has not been blocked as might appear on the fracture table where the tendency for varus malreduction may be apparent. Also, this position allows for sufficient working space for inserting the nail. In the lateral position, the distal fragment should be ‘flexed’ in accordance with the flexion of the proximal fragment by the iliopsoas to obtain a sagittal plane reduction. This position allows the guide pin and nail to be inserted without interference by the torso (Figure 6).

Both the piriformis fossa and trochanteric entry nail can be used with the lateral position [9-10]. However, approaching the trochanteric entry point appears to be more feasible in OA/STF with a shortened neck since the anatomical relationship between the greater trochanter and femoral shaft is usually normal. Access to the entry point is easy and varus malreduction can be avoided. The lateral position also facilitates sagittal plane reduction [3,9-10], allows the avoidance of complications caused by the fracture table [9-10], and improves accessibility to the assistant [9]. Although manual traction by an assistant is usually successful [9-10], the Shantz screw, a femoral distractor, or limited open reduction is occasionally required [3,9-10]. Rotational reduction can be achieved by rotating the leg and can be assured by a comparison of the true anteroposterior and lateral C-arm images of the uninjured side [9]. Some tricks for the C-arm setting to display accurate anteroposterior and lateral images of the proximal and distal femur should be noted [9-10]. The insertion of distal interlocking can be accomplished by adjusting the C-arm.

## Conclusions

In intramedullary nailing for subtrochanteric fractures of the femur, varus malreduction must be avoided to prevent nonunion. If the fracture is accompanied by pre-existing osteoarthritis of the ipsilateral hip joint, the entry point will inevitably become misplaced laterally and result in varus, especially when a fracture table with a perineal post is used. In such cases, the lateral decubitus position provides easy access to the entry point and allows accurate reduction.
